# A Molecular Dynamics Study on Mechanical and Tribological Properties of Polyimide Modified with Graphene: Size and Layer Effects

**DOI:** 10.3390/polym18151816

**Published:** 2026-07-24

**Authors:** Yangyang Chen, Song Yuan, Hongtao Liu

**Affiliations:** 1 Jiangsu XCMG Construction Machinery Research Institute Co., Ltd., Xuzhou 221004, China; yangyangchen1@163.com (Y.C.);; 2 School of Materials Science and Physics, China University of Mining and Technology, Xuzhou 221004, China

**Keywords:** modified polyimide, molecular dynamics, graphene layers, graphene size effects, tribological properties, mechanical properties

## Abstract

Graphene, with excellent mechanical and self-lubricating properties for polymer modification, can be single- or multi-layered (3–10 layers). In this study, molecular dynamics simulations have been employed to qualitatively explore the relative trends and internal modification mechanism of polyimide (PI) modification by single-layer graphene and three-layer graphene with different sizes. Small-sized single-layer graphene (SSLG), small-sized multi-layer graphene (SMLG), large-sized single-layer graphene (LSLG), and large-sized multi-layer graphene (LMLG) were introduced into the PI matrix at an identical mass fraction with initially uniform dispersion during model construction. The tensile mechanical and frictional behaviors of graphene-modified PI were systematically examined. The results indicate that graphene addition effectively improves both the mechanical and tribological properties of PI. At a fixed filler mass fraction, SSLG exhibits the strongest interaction with PI, with a binding energy of 396.8 kJ/mol. The fractional free volume of SSLG-reinforced PI reaches 15.3%, which is considerably lower than the value calculated for pure PI (20.3%). The average elastic modulus of the SSLG-modified PI is 70.4% higher than that of pure PI, an increase which exceeds that of the SMLG-modified PI (45.2%), LSLG-modified PI (26.5%), and LMLG-modified PI (14.0%). In terms of tribological properties, the SMLG-modified PI exhibits optimal friction with an average friction coefficient of 0.105, which is 48.3% lower than that of pure PI and lower than the values for the SSLG (0.138), LSLG (0.156), and LMLG (0.182) systems. This work mainly draws qualitative structure-property rules and provides key theoretical fundamentals and design principles for tailoring the mechanical and tribological performance of high-performance graphene-reinforced polyimide composites.

## 1. Introduction

Thermoplastic polyimide (PI) is widely employed in the fabrication of bearings, gears, and various wear-resistant components due to its high-temperature resistance, low-friction coefficient, high mechanical strength, and superior chemical stability, minimizing material loss caused by frictional wear [1,2,3,4]. However, the frictional and mechanical deficiencies of pure PI materials in practice limit applications under more severe operating conditions [5,6,7]. Research studies have indicated that the incorporation of appropriate modifiers and the rational design of PI composites can lead to significant improvements in both tribological performance and mechanical properties [8,9].

Graphene is a two-dimensional carbon nanomaterial with a hexagonal honeycomb crystal lattice structure, excellent mechanical properties, and self-lubricating characteristics that can be employed to reinforce polymers [10,11,12]. This has led to its widespread use as a nanofiller in polymer matrices for developing high-strength, low-friction composites. A number of studies have been conducted using molecular dynamics simulations and experimental approaches to gain an understanding of the graphene strengthening mechanisms. Chen et al. [13] have found that the use of mini-sized sheets (300 × 400 nm) of graphene to fill polyurethane resulted in improved performance, notably with respect to mechanical properties, electrical conductivity, thermal conductivity, thermal stability and flame retardance. Hwang et al. [14] have found that incorporating graphene as a composite filler in the PI matrix increases the initial PI modulus from 2.63 GPa to 4.43 GPa. Chai et al. [15] constructed PI composite models with fluorinated graphene (FG) contents of 0 wt%, 1 wt%, 3 wt%, and 5 wt%. The composite modified with 1 wt% FG exhibits a 77% increase in Young’s modulus when compared with pure PI, achieving the optimal mechanical reinforcement. The composite filled with 3 wt% FG shows the highest interfacial bonding energy, forming the most stable lubricating film, and the lowest friction coefficient (approximately 0.08). In practical applications, graphene incorporation can be classified as single- or multi-layered [16,17]. Multi-layer graphene is formed by stacking 3 to 10 layers [17]. The reported studies of graphene-modified composite materials have largely focused on the reinforcing effects of the graphene content and size with respect to polymer materials. A possible synergistic enhancement and regulation of the mechanical and tribological properties by varying the size and number of graphene layers has remained unexplored. The role of size and layer number in influencing the reinforcement and overall efficacy of polyimide composites is not well understood and warrants a systematic investigation.

A molecular dynamics simulation enables an effective evaluation of material properties, the characterization of damage evolution, and analysis of property enhancement mechanisms. It provides a multiscale perspective that serves to connect macroscopic friction, wear, and mechanical performance with phenomena at molecular and atomic scales [18]. Molecular dynamics simulation is widely applied as a reliable approach for investigating the mechanical and tribological properties of materials. Li et al. [19] have reported a molecular dynamics simulation of the changes in the tribological and mechanical properties of graphene-reinforced polyethylene composites during friction. Moreover, Bamane et al. [20] employed molecular dynamics simulations to evaluate the effects of various functionalized graphene quantum dots (GQDs) on the mechanical properties of materials. The results demonstrate that amine-functionalized GQDs increase the Young’s modulus and yield strength of the epoxy resin by 14% and 47%, respectively. Zhang et al. [21] investigated the effects of nano-diamond-decorated reduced graphene oxide and other fillers on the molecular-scale frictional properties of PI. Although relevant simulations have been conducted on the modification of graphene in composite materials, the inherent microscopic mechanism that underpins graphene layer number and size effects with respect to the mechanical and tribological properties of polyimide composites has not been examined in depth.

In this study, molecular dynamics models of graphene-PI composites prepared under melt-blending conditions were constructed to qualitatively investigate how four combined graphene morphologies, defined by flake size and layer number, affect the mechanical and tribological properties of the PI matrix at a fixed filler mass fraction. This study provides vital theoretical support and structural design guidance for the precise regulation of the friction coefficient and mechanical properties of high-performance graphene-reinforced polyimide composites. The optimized composites can address the failure of load-bearing components under cyclic heavy loads, reduce energy consumption, and prolong the service life of friction pairs, thereby overcoming the performance bottlenecks of neat PI in industrial applications. The proposed molecular dynamics model and simulation framework are universal and applicable to most thermoplastic polymers, such as polyurethane, polyether ether ketone, polyetherimide, polysulfone, and polyaryletherketone.

## 2. Simulation

### 2.1. Model Construction

Molecular simulation software typically operates at the nanometer length scale as it is designed to model the behavior of atoms and molecules. However, graphene sheets used in practical applications [17] generally have diameters at the micrometer scale. To enable simulations of graphene-dispersed reinforced PI polymers under controlled molecular-level conditions, reduced graphene sheet sizes were used to construct scaled-down models. The modeling strategy adopted in this work is consistent with that reported in references [15,19,20,21], which verifies the wide recognition of this scaled-down modeling method within the research community. In this situation, the results cannot be quantitatively transferred to real micron-sized sheets. Nevertheless, the selected graphene sizes were designed to compare the relative effects of graphene size and layer number on the tribological and mechanical properties of PI materials. The qualitative trends observed from these comparisons are physically meaningful and may provide useful guidance for larger-scale systems.

**Figure 1:**
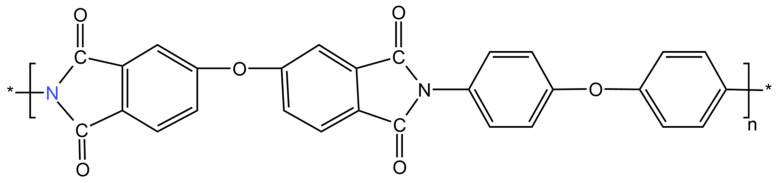
Chemical structure of PI.

**Figure 2:**
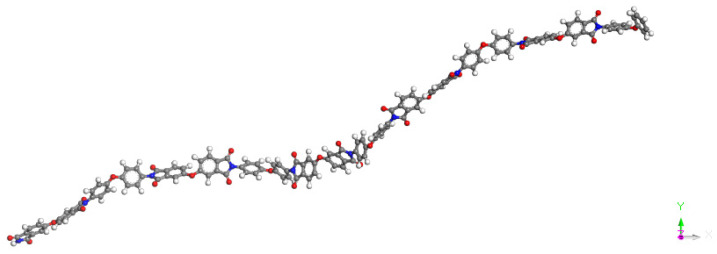
PI molecular chain structure with a polymerization degree of 5.

**Figure 3:**
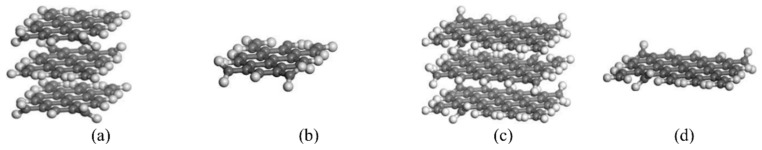
Graphene models with different sizes and layers. (**a**) SMLG; (**b**) SSLG; (**c**) LMLG; (**d**) LSLG.

A PI molecular chain model with a polymerization degree of 5 was constructed from PI monomers; the associated molecular structure is shown in Figure 1, with the chain structure presented in Figure 2. Four graphene models with different sizes and layer numbers were constructed and are denoted as small-sized single-layer graphene (SSLG), large-sized single-layer graphene (LSLG), small-sized multi-layer graphene (SMLG), and large-sized multi-layer graphene (LMLG). The dimensions of SSLG and SMLG were set at 4.92 Å × 8.52 Å, whereas those of LSLG and LMLG are defined as 9.84 Å × 8.52 Å. Both SMLG and LMLG consist of three graphene layers with an interlayer spacing of 3.4 Å. It should be noted that the multi-layer graphene system refers to a three-layer graphene system. To eliminate edge effects arising from unsaturated bonds, hydrogen termination was applied to all graphene sheets to remove unsaturated carbon atoms at graphene edges, reduce the chemical activity of edge atoms, and partially mitigate the interference of varying edge atom density on interfacial interactions. The corresponding structures of LSLG, SSLG, SMLG and LMLG are shown in Figure 3.

**Figure 4:**
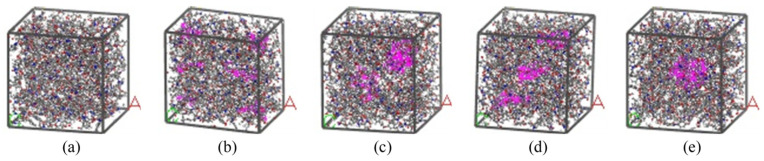
Models of pure PI and PI modified with graphene of different size and number of layers. (**a**) Pure PI; (**b**) PI modified with SSLG; (**c**) PI modified with SMLG; (**d**) PI modified with LSLG; (**e**) PI modified with LMLG.

In order to simulate the effect of graphene on the mechanical properties of PI, both pure PI and graphene-modified PI models were constructed. A periodic cubic lattice with dimensions of 4 nm × 4 nm × 4 nm was first established. This periodic unit cell setup eliminates surface and edge effects. The PI molecular chains were packed into this lattice at a density of 1.35 g/cm^3^ using the Bias Monte Carlo method. Five distinct models were designed: model 1, pure PI without graphene (Figure 4a); model 2, PI filled with SSLG (Figure 4b); model 3, PI filled with SMLG (Figure 4c); model 4, PI filled with LSLG (Figure 4d); and model 5, PI filled with LMLG (Figure 4e). It should be noted that the mass fraction of graphene added in all modified PI models was uniformly controlled at 3%. The modeling framework was constructed for graphene-PI composites prepared under melt-blending conditions, aiming to separately reveal the synergistic effects of graphene lateral size and stacking layer number on the mechanical and tribological performance of the PI matrix under identical filler loading. Fixing the graphene mass fraction at 3 wt% eliminates the confounding variable of filler content, allowing us to isolate the intrinsic property differences induced by graphene morphology rather than loading dosage. Single-layer graphene and three-layer graphene possess distinct mass per sheet, so adjusting the sheet count in each composite system is the only feasible strategy to maintain equal total graphene mass across all four filled groups. To achieve this constant mass fraction, different numbers of graphene sheets were used in each system: six SSLG sheets for SSLG/PI, two SMLG sheets for SMLG/PI, three LSLG sheets for LSLG/PI, and one LMLG sheet for LMLG/PI.

To reproduce an ideal state after sufficient melt blending of graphene and polyimide, graphene sheets were arranged in an ideally uniform, agglomeration-free distribution throughout the unit cell at the initial modeling stage, which closely mimicked the homogeneous dispersion of graphene at the same mass fraction under ideal preparation conditions. Objectively, for graphene fillers with equal loading mass under ideal homogeneous dispersion, small-sized single-layer graphene inherently possesses a greater number of sheets, a larger total interfacial contact area, higher edge atom density, better dispersion uniformity, and a shorter average distance to the friction interface. All graphene sheets were uniformly distributed in the 4 nm × 4 nm × 4 nm unit cell during the initial packing process. During the equilibration stage, graphene sheets were allowed to translate, rotate, and relax freely within the polyimide matrix. Driven by van der Waals interactions between graphene and PI molecular chains, graphene sheets spontaneously rearranged into random disordered distributions throughout the relaxation process. The final dispersion morphology and the distance between graphene fillers and the friction interface after equilibration are intrinsically governed by the inherent thermodynamic interactions of each system.

Geometry optimization and molecular dynamics equilibrium processes were performed to establish the global and local minimum energy configurations. Firstly, the five models were geometrically optimized using the smart algorithm within the Forcite module. Subsequently, ten cycles of annealing were conducted over the temperature range of 300–500 K, applying the NVT ensemble (constant number of particles, volume, and temperature) to identify the configuration with the lowest local energy. The annealing temperature range (300–500 K) approximates the room-temperature service condition of the material, enabling a mild system relaxation, eliminating internal stress and regularization of molecular chain conformations. This also circumvents structural degradation of the matrix caused by high temperatures. The models were then subjected to sequential molecular dynamics equilibrium optimizations applying the NVT ensembles with a time step of 1 fs and a total duration of 2 ns for each ensemble to eliminate residual stresses. The temperature was set at 300 K and the pressure at 0.1 MPa throughout the processes, which conforms to the actual service environment of the material. This ensures full relaxation of molecular chains and the smooth attainment of thermodynamic equilibrium, avoiding structural distortion caused by a deviation of temperature and pressure from actual working conditions, and guaranteeing the reliability of subsequent simulations of mechanical and tribological properties. All optimization calculations were performed using the COMPASS II force field, an extensively validated classical force field widely adopted for graphene-polymer composite simulations [15,18,19]. The COMPASS II force field enables high-precision simulation calculations for a wide variety of systems, including organic compounds, polymers, gaseous molecules, metals, metal oxides, metal halides, and zeolites. Furthermore, it contains dedicated parameters for aromatic imide groups and sp2-hybridized carbon atoms, both of which are essential for accurately characterizing the interfacial interactions between PI and graphene [18]. The van der Waals terms were calculated using the atom-based method, whereas the electrostatic terms were evaluated using the Ewald method. Periodic boundary conditions were applied, and a cutoff distance of 1.25 nm was set for the molecular potential energy calculations.

To investigate the effect of graphene on the tribological properties of PI composites, a three-layer tribological model was established, consisting of upper and lower iron (Fe) atomic layers and an optimized PI lattice as the middle layer. The method for constructing the tribological model is based on research reported by Chai et al. [15] and Li et al. [19]. The Fe atomic layers had dimensions of 4 nm × 4 nm × 1.1 nm, whereas the middle layer comprised the PI model that had undergone geometric optimization, annealing, and dynamic equilibrium optimization. The density of the model after structural optimization was consistent with the theoretical density (1.35 g/cm^3^).

All molecular dynamics simulations were performed in triplicate. The final results are expressed as a mean. Any error is associated with thermal fluctuation of atoms and statistical calculation, which were addressed by applying sufficient system relaxation and multi-frame data averaging.

As an enlarged-cell robustness check, supplementary simulations were performed using a 5.5 × 5.5 × 5.5 nm^3^ unit cell with approximately 2.6 times the volume of the original 4 nm cell. The dimensions of SSLG and SMLG were set at 7.38 Å × 12.78 Å, whereas those of LSLG and LMLG were defined as 14.38 Å × 12.78 Å. SSLG and LSLG were single-layer graphene sheets, whereas SMLG and LMLG consisted of three layers. The PI matrix was packed at a fixed density of 1.35 g/cm^3^, and the graphene weight fraction was maintained at 2.6% in all composites. Accordingly, the unit cells contained six SSLG sheets for SSLG/PI, two SMLG sheets for SMLG/PI, three LSLG sheets for LSLG/PI, and one LMLG sheet for LMLG/PI. All simulation parameters, including the COMPASS II force field, annealing and equilibration protocols, tensile loading conditions, and friction shear settings, were kept consistent with those of the original model. Compared with pure PI, the average elastic moduli of PI composites filled with SSLG, SMLG, LSLG, and LMLG increased by 65.8%, 48.7%, 24.8%, and 11.8%, respectively, while their friction coefficients decreased by 22.6%, 38.5%, 18.4%, and 9.5%, respectively. In the enlarged cell, the graphene edge atom density was reduced, the available filler-dispersion space was increased, and the average filler-friction interface distance distribution changed. Nevertheless, the relative performance ranking of all material groups remained consistent with that obtained using the 4 nm cell. This enlarged-cell robustness check therefore supports the robustness of the qualitative trends observed in the original model, particularly the rankings of the reinforcing and lubricating performance of the four graphene fillers, under moderate changes in cell size, edge atom density, and filler spatial distribution.

### 2.2. Simulation of Mechanical Properties

The mechanical properties of the model after dynamic equilibrium were calculated using the constant strain method. Specifically, four strains (*ε* = −0.003, −0.0015, 0.0015, 0.003) were applied separately to the model along six directions (*X*, *Y*, *Z*, *XY*, *XZ*, *YZ*). Based on the definition of virial stress, the stress components of the composite material in each direction can be calculated. The calculations of the elastic modulus are given in Equations (1)–(3).

*E_i_* = *σ_i_/ε_i_* (*i* = 1, 2, 3) (1)

*σ_i_ = **C**_ij_ε_j_* (*i*, *j* = 1, 2, 3, …,6) (2)

*ε_i_* = ***S**_ij_σ_j_* (*i*, *j* = 1, 2, 3, …,6) (3)

where *σ_i_* represents the stress; *ε_i_* is the strain; *C_ij_* denotes the stiffness matrix; *S_ij_* is the compliance matrix; *E*_1_, *E*_2_, and *E*_3_ represent the elastic moduli along the *X*, *Y*, and *Z* directions, respectively; *E*_max_ denotes the maximum elastic modulus.

The calculation of the bulk modulus and shear modulus for the composite material system is presented in Equations (4)–(9).

*B*_V_ = [*C*_11_ + *C*_22_ + *C*_33_ + 2*C*_12_ + 2*C*_13_ + 2*C*_23_]/9 (4)

*G*_V_ = [*C*_11_ + *C*_12_ + *C*_33_ + 3*C*_44_ + 3*C*_55_ + 3*C*_66_ − *C*_12_ − *C*_13_ − *C*_23_]/15 (5)

*B*_R_ = 1/(*S*_11_ + *S*_22_ + *S*_33_ + 2*S*_12_ + 2*S*_23_) (6)

*G*_R_ = 15/[4(*S*_11_ + *S*_22_ + *S*_33_ − *S*_12_ − *S*_13_ − *S*_23_) + 3(*S*_44_ + *S*_55_ + *S*_66_)] (7)

*B*_H_ = [*B*_V_ + *B*_R_]/2 (8)

*G*_H_ = [*G*_V_ + *G*_R_]/2 (9)

where *B*_H_, *B*_R_ and *B*_V_ represent the actual bulk modulus, Reuss bulk modulus and Voigt bulk modulus, respectively; *G*_H_, *G*_R_ and *G*_V_ are the actual shear modulus, Reuss shear modulus and Voigt shear modulus, respectively.

### 2.3. Simulation of Tensile Properties

The optimized model was continuously stretched along the Y-direction at a constant stress increment of 0.05 GPa per step with a total of six steps to reach a final stress of 0.3 GPa, which falls within the elastic deformation range of the composite, ensuring that the material maintains an intact molecular chain and interfacial structure without irreversible plastic deformation, structural damage or bond fracture during stretching. After each stretching step, a geometry optimization and 100 ps molecular dynamics simulation were performed to allow the atoms in the system to achieve dynamic equilibrium according to the dimensions of the stretched model, simulating the creep behavior of polymer chains that applies in actual tensile processes. Upon completion of stretching, the strains under different stresses were calculated separately to obtain the polymer stress-strain curve.

### 2.4. Simulation of Friction Properties

After undergoing geometry optimization, annealing, and dynamic equilibrium, the molecular friction model for the polymer-metal sliding pair was subjected to tribological property simulation using the Confined Shear function in the Forcite module at a specified force field, temperature, load, velocity, and time parameters. During the friction process, a normal pressure of 0.05 GPa and a relative sliding velocity of 0.5 Å/ps were applied to the Fe atomic layer, which falls within the conventional load range for polymer-metal tribological simulations. The simulation was conducted at 300 K (room temperature), with a total simulation time of 80 ps, a step count of 80,000, and a time step of 1 fs. The vertical system pressure (*P*) and horizontal force (*f*) between the Fe metal and PI polymer during friction were extracted from the model. The friction coefficient (*μ* = *f*/*P*) was calculated separately, and the changes in temperature and energy during friction were analyzed.

## 3. Results and Discussion

### 3.1. Mechanical Properties

The elastic modulus, bulk modulus, and shear modulus of PI and PI polymers reinforced with graphene of different sizes and layers at the same graphene content (3%) were calculated separately, and the results are presented in Table 1. It is evident that the incorporation of graphene effectively enhances the elastic modulus, bulk modulus, and shear modulus. This simulation result is consistent with the experimental findings reported by Dai et al. [22], where incorporating 11 wt% graphene in PI served to increase the modulus from 2.01 GPa to 4.04 GPa. It should be noted that SSLG inclusion delivers the best reinforcement effect. When compared with pure PI, the average elastic modulus, actual bulk modulus, and actual shear modulus of PI modified with SSLG are increased by 70.4%, 87.6%, and 42.4%, respectively. In contrast, PI modified with LMLG shows the lowest level of reinforcement, with an increase of 14.0% in the average elastic modulus, 17.4% in the actual bulk modulus, and 9.9% in the actual shear modulus. In the case of PI modified with LSLG and SMLG, an increase in average elastic modulus of 26.5% and 45.2%, respectively, is observed; the associated increases in the actual bulk modulus are 24.8% and 80.2%, and 12.1% and 15.2% for the actual shear modulus.

The average elastic modulus of pure PI obtained from simulations (3.21 GPa) is consistent with the value (3.206 GPa) measured in nanoindentation experiments (indentation depth: 1 μm), as reported by Ji et al. [23]. The simulated elastic modulus, bulk modulus, and shear modulus of pure PI are close to the values reported by Chai et al. [15], with relative errors of approximately 2.5% for the average elastic modulus and 3% for the shear modulus. The morphology-dependent trend resulting from the combined variation in flake size and layer number is consistent with the experimental results reported by other researchers. Patra et al. [24] prepared polypropylene composites by melt compounding with three grades of H-type graphene nanoplatelets with an identical thickness of 15 nm, including small-sized H5 (5 μm), medium-sized H15 (15 μm), and large-sized H25 (25 μm). Their results revealed that the tensile strength and elastic modulus increased as the lateral size of graphene nanoplatelets decreased. Maldonado-Magnere et al. [25] fabricated fluororubber composites via solution blending using two types of multi-layer graphene nanoplatelets with different lateral sizes: small-sized GN5 (5 μm) and large-sized GN15 (15 μm). Their results verified that, at the same filler loading, small-sized graphene nanoplatelets exhibited better dispersion and stronger interfacial bonding with the polymer matrix, thereby showing a markedly stronger mechanical reinforcement effect than their large-sized counterparts.

**Figure 5:**
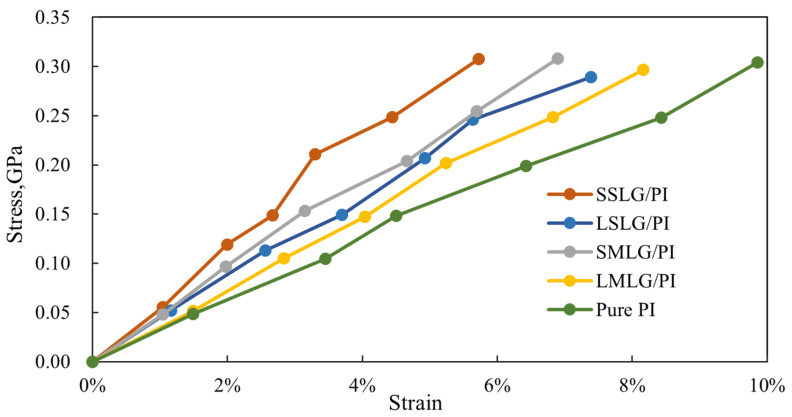
Stress-strain curves for PI and PI modified with SSLG, LSLG, SMLG, and LMLG.

**Figure 6:**
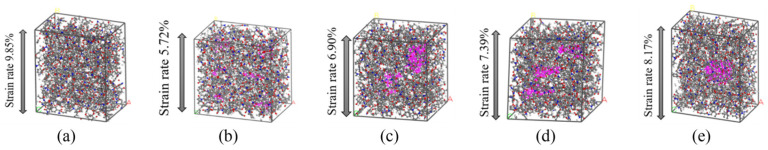
Molecular lattices of pure PI and graphene-modified PI composites after tensile loading at 0.3 GPa applied stress. (**a**) Pure PI; (**b**) PI modified with SSLG; (**c**) PI modified with SMLG; (**d**) PI modified with LSLG; (**e**) PI modified with LMLG.

The mechanical properties of graphene-reinforced PI were further assessed in tensile simulations carried out on pure PI and modified PI; the resultant stress-strain curves are shown in Figure 5. The elastic modulus calculated from the slope of the stress-strain curve obtained from tensile simulations is essentially equivalent to that derived from the constant stress method, with an error of ±8%. (Table 2). As shown in Figure 6, the PI molecular chains in pure PI and graphene-modified PI composites, following tensile loading at 0.3 GPa applied stress, are elongated. Pure PI (Figure 6a) exhibits the most severe deformation, with a maximum strain of 9.85%, whereas the graphene-modified PI composites show a significantly reduced deformation. The SSLG/PI system (Figure 6b) exhibits the smallest strain (5.72%) when compared with SMLG/PI (6.90%, Figure 6c), LSLG/PI (7.39%, Figure 6d), and LMLG/PI (8.17%, Figure 6e), which is consistent with the trend observed for elastic modulus enhancement.

To further elucidate the interfacial stress distribution and load transfer mechanism between graphene fillers and the PI matrix, we conducted phase-separated virial stress analysis using the trajectories obtained from tensile molecular dynamics simulations. The atomic system was partitioned into two phase groups corresponding to the PI matrix and graphene reinforcement. After the tensile simulation, post-processing was carried out using the Forcite Analysis module. Trajectory frames captured at the intermediate tensile stage under 0.15 GPa were extracted to calculate the average virial stress of the two phases for all four composite systems.

**Figure 7:**
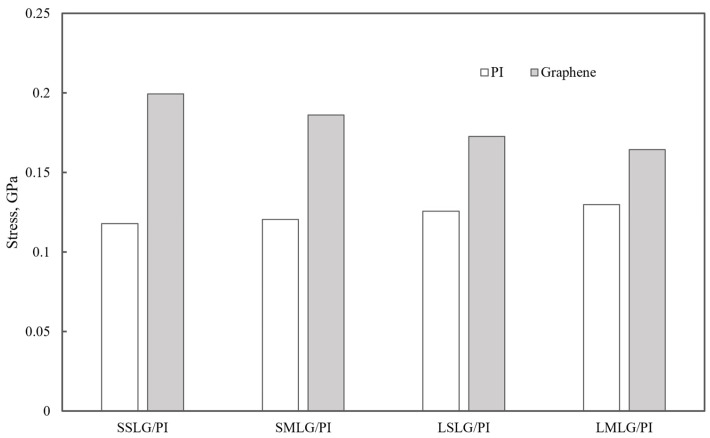
The average stress of the PI matrix and graphene reinforcement in SSLG/PI, LSLG/PI, SMLG/PI, and LMLG/PI composites at the tensile load of 0.15 GPa.

Figure 7 compares the average stress of the PI matrix and graphene reinforcement in SSLG/PI, LSLG/PI, SMLG/PI, and LMLG/PI composites at a tensile load of 0.15 GPa. In all modified systems, graphene carries substantially higher average stress than the PI matrix. This confirms that external stress can be efficiently transferred from the low-modulus PI matrix to high-strength graphene through interfacial van der Waals forces. Among all samples, the SSLG/PI composite achieves the highest graphene-to-matrix stress ratio of 1.69, reflecting the most efficient interfacial load transfer. This finding is in excellent agreement with its maximum interfacial binding energy, minimum fractional free volume, and greatest improvement in elastic modulus. The stress ratios of SMLG/PI, LSLG/PI, and LMLG/PI progressively decrease to 1.55, 1.37, and 1.20, respectively, which follows the decreasing trend of the composite elastic modulus. The combined morphology defined by graphene flake size and layer number strongly affects the interfacial interaction and load-transfer efficiency between graphene and the PI matrix and, in turn, the macroscopic mechanical properties of the composites. At a fixed filler mass fraction, graphene with the small-size, single-layer morphology exhibits more efficient interfacial stress transfer than the other three combined morphologies examined here. The observed performance differences therefore reflect the combined effects of flake size and layer number rather than either variable in isolation.

During the stretching of the modified PI composites, the PI molecular chains interact with the graphene surface via van der Waals forces and electrostatic interactions. When the molecular system is subjected to external stress, the intermolecular forces between graphene and PI exert a reinforcing effect, enabling the transfer of stress from the PI molecular chains to the graphene. This facilitates efficient load transfer, bridges microcracks, delays crack propagation, and enhances interfacial adhesion. As graphene exhibits higher strength and superior deformation resistance than PI under the same stress, it can effectively inhibit crack propagation [26]. As a result, graphene-modified PI composites demonstrate significantly reduced deformation under tensile loading. The simulation results indicate that, at a fixed graphene content, the mechanical strength of the composites is positively correlated with the uniformity of graphene dispersion. SSLG (six small single-layer sheets) exhibits the most uniform dispersion and the largest interfacial contact area, enabling efficient stress transfer from PI to graphene. SMLG (two small three-layer sheets) shows good dispersion, but its stacked structure reduces the effective contact area per unit mass, resulting in slightly weaker reinforcement. In contrast, LSLG (three large sheets) has larger inter-sheet distances, which reduce stress transfer efficiency and mechanical enhancement. Consequently, the small-size morphologies (SSLG and SMLG) produce a more pronounced reinforcing effect than the large-size morphologies (LSLG and LMLG) at the same filler loading.

This finding provides a clear theoretical basis for the design of high-strength PI composite materials. The SSLG/PI system with outstanding mechanical performance is particularly suitable for manufacturing heavy-duty gears, hydraulic seals, and aerospace structural supports, which require high stiffness and anti-deformation capability to withstand long-term harsh operating conditions.

### 3.2. Binding Energy of PI and Graphene

**Figure 8:**
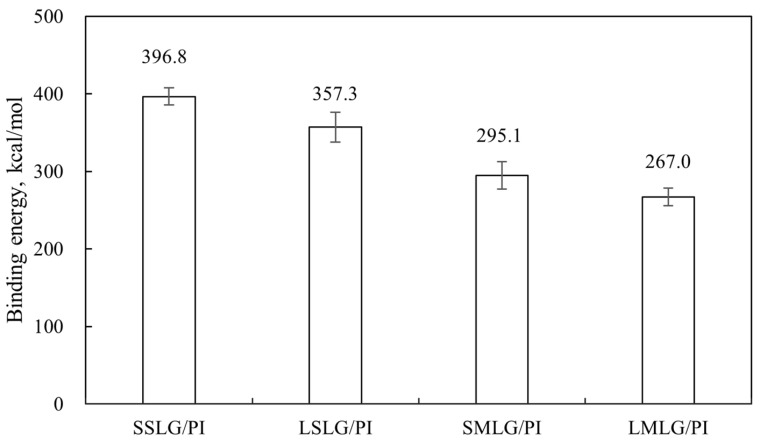
The binding energies of PI and graphene for the four systems. Error bars represent the standard deviation (SD) of three parallel simulations.

The binding energies between graphene with different sizes and PI were considered. Binding energy reflects the compatibility of different components in a system. The binding energy was calculated using Equation (10);

(10) $$E_{bind}=-(E_{total}-E_{PI}-E_{g})$$

where *E*_bind_ represents the binding energy, *E*_total_ is the total energy of the system at equilibrium, and *E*_PI_ and *E*_g_ denote the energies of the PI and graphene, respectively. The results are given in Figure 8. It can be seen that SSLG exhibits the highest binding energy with PI, reaching 396.8 kJ/mol. The SSLG has a larger relative contact area with PI molecules, which facilitates interaction and improved dispersion with PI. The consequent increase in effective reinforcement interfaces serves to significantly enhance the mechanical properties of the composite. In contrast, LMLG has a smaller relative contact area with PI, resulting in the lowest binding energy (267.0 kJ/mol), which is 32.7% lower than SSLG. The binding energies of LSLG and SMLG with PI are 357.3 kJ/mol and 295.1 kJ/mol, respectively.

### 3.3. Fractional Free Volume of PI and PI Modified with Graphene

Applying the free volume theory and the Fox-Flory molecular mobility theory [27], the molecular unit cell consists of two distinct regions: the volume occupied by molecular chains (*V*_o_) and the holes (*V*_f_). The size of these holes determines the magnitude of the fractional free volume (FFV). The occurrence of the latter enables the free movement of molecular chains, while also governing material density, mechanical and tribological properties. The FFV was calculated using Equation (11):

(11) $$FFV=\frac{V_{f}}{V_{f}+V_{o}}$$

**Figure 9:**
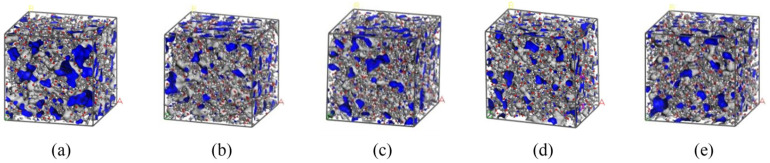
Fractional free volume of pure PI and PI modified with graphene. (**a**) Pure PI; (**b**) SSLG/PI; (**c**) SMLG/PI; (**d**) LSLG/PI; (**e**) LMLG/PI. Note: the blue area represents free volume voids.

**Figure 10:**
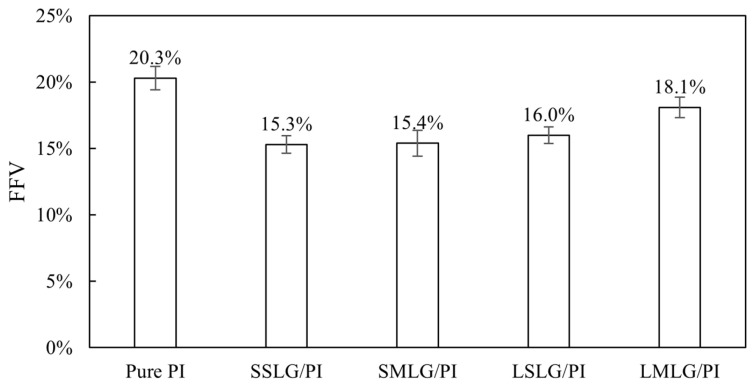
The calculated FFV of pure PI and the four graphene-modified PI systems. Error bars represent the SD of three parallel simulations.

The FFV of pure PI and graphene-modified PI composites was analyzed to account for the variations in density illustrated in Figure 9. As shown in Figure 10, the pure PI model exhibits a relatively large free volume with an FFV of 20.3%. Following graphene incorporation, the PI molecular chains interact with the graphene nanofillers, eliminating large voids in the unit cell and resulting in a more compact structure with a significant reduction in FFV, which is consistent with the study reported by Li et al. [19]. The SSLG/PI system exhibits the greatest contact area between SSLG and the PI molecular chains, resulting in the lowest FFV (15.3%). This arises from the larger interfacial contact and stronger interfacial adhesion between SSLG and PI chains; van der Waals attractive forces enable tighter molecular packing. The FFV values of the SMLG/PI, LSLG/PI, and LMLG/PI composites increase in order: 15.4%, 16.0%, and 18.1%.

### 3.4. Friction Performance

The friction coefficients of PI and the four PI composite systems were also calculated. The relative molecular concentration distribution and temperature distribution along the thickness direction were analyzed from the trajectory files. The underpinning mechanisms contributing to enhanced PI tribological properties by graphene were considered.

The calculated friction coefficients are presented in Table 3. Pure PI exhibits the highest average friction coefficient (0.203), while the average friction coefficients of the PI composites modified with SSLG, LSLG, SMLG and LMLG are 0.138, 0.156, 0.105 and 0.182, corresponding to reductions of 32.0%, 23.2%, 48.3% and 10.3% relative to pure PI, respectively. The simulation results demonstrate that the incorporation of graphene can significantly improve the tribological properties of PI. This is consistent with the experimental findings reported by Ruan et al. [28], where the average friction coefficients of pure PI and unmodified graphene-reinforced PI composites were 0.34 and 0.26, respectively, under dry sliding conditions against a GCr15 steel ring (applied load: 15 N, sliding speed: 1.04 m/s). The simulated friction coefficients are lower than the experimental values, which may be attributed to the differences between the idealized simulation model and the experimental conditions. In molecular dynamics simulations, the friction interface is constructed as an ideal, smooth, and defect-free contact surface, which does not take into account surface roughness, impurity adsorption, and local stress concentration associated with experimental testing. It should be noted that the simulated friction coefficient of pure PI (0.203) is close to the value (0.234) reported by Chai et al. [15], with a relative error of 15%.

**Figure 11:**
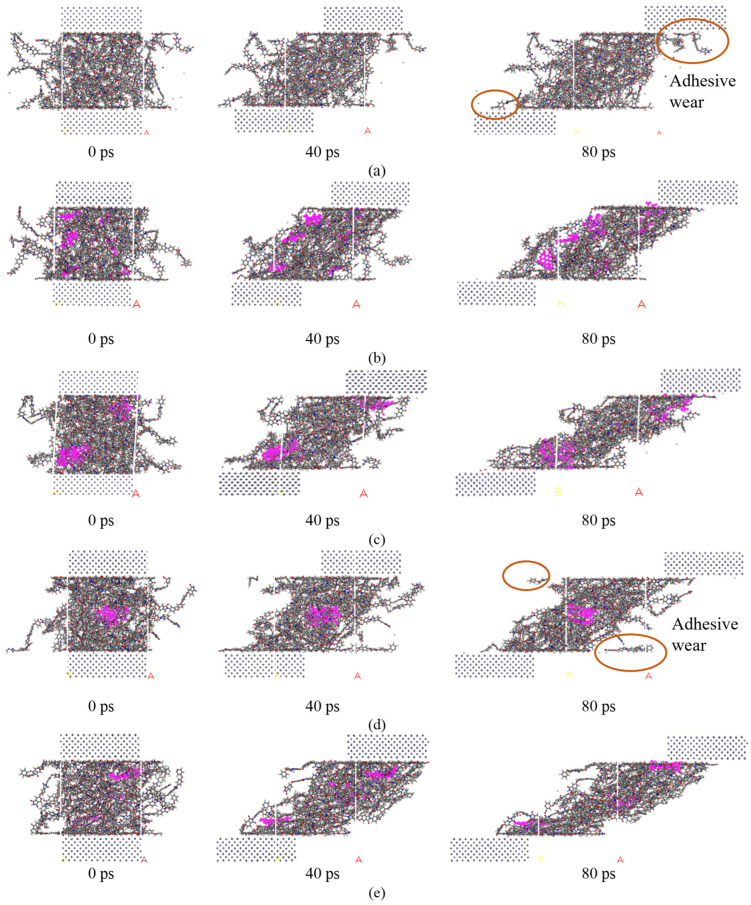
Snapshots of PI and PI modified with graphene. (**a**) Pure PI; (**b**) SSLG/PI; (**c**) SMLG/PI; (**d**) LMLG/PI; (**e**) LSLG/PI.

Molecular dynamics simulation snapshots of pure PI and graphene-modified PI composites at different shear times (0 ps, 40 ps, and 80 ps) are presented in Figure 11, where the purple regions represent the graphene nanofillers. The molecular dynamics snapshots serve to visually verify the mechanism by which graphene size and layer number regulate the tribological properties of the PI composites, which is consistent with the calculated friction coefficients. In the case of pure PI (Figure 11a), the friction interface is dominated by direct contact and shearing between Fe atoms and the PI molecular chains. Under continuous shear stress, the PI chains deform, entangle, and even fracture at the interface. Distinct detachment of surface PI molecules from the matrix is observed throughout the sliding process, which is a typical microscopic characteristic of adhesive wear. This behavior results in high interfacial shear resistance and the highest friction coefficient (0.203).

In contrast, graphene-reinforced PI systems (Figure 11b,c,e) exhibit distinctly different interfacial behavior: graphene flakes migrate to the friction interface during shearing and act as a solid lubricant, transforming the friction pair from Fe-PI to Fe-graphene, which significantly reduces shear resistance and improves tribological performance. After sliding across the full lattice within 80 ps, the friction surfaces of these three composites remain intact without obvious morphological changes or molecular detachment, indicating effective suppression of adhesive wear by graphene fillers. Different from the above three systems, LMLG/PI (Figure 11d) cannot form a continuous and dense lubricating film at the friction interface due to the large size and poor mobility of large-sized multi-layer graphene. Direct contact between Fe and PI still dominates the sliding process. Similar to pure PI, obvious detachment of surface PI molecules is captured in the LMLG/PI system over the 80 ps simulation period, leading to noticeable adhesive wear and a relatively high friction coefficient of 0.182.

At the same graphene content, the SMLG/PI composite shows the lowest friction coefficient (0.105) with distinctly higher values for SSLG/PI, LSLG/PI and LMLG/PI. This result is consistent with the experimental findings reported by Kong et al. [29]. In their study, Kong and co-workers dispersed hydrothermally reduced graphene oxide (rGO) into PAO4 base oil and investigated three types of rGO samples: G2 (few-layer rGO, thickness: 3–4 nm, lateral size: ~5 μm), G10S (small-sized multi-layer rGO, thickness: 10–12 nm, lateral size: 0–4 μm), and G10L (large-sized multi-layer rGO, thickness: 10–12 nm, lateral size: 4–10 μm). At a concentration of 0.1 mg/mL, G10S delivered the most prominent friction reduction of 37%, compared with 22% for G2 and 19% for G10L. The central trend, namely that the small-size, multi-layer morphology provides the greatest friction reduction whereas the large-size, multi-layer morphology provides the least, is consistent with our simulation results. In addition, as shown in Figure 11, at a fixed filler content, the small-size morphologies (SMLG and SSLG) are more likely to reach the friction interface because their smaller flakes facilitate transport and participation in the tribological response (Figure 11b,c), potentially promoting the formation of a lubricating film. This serves to transform the friction pair from Fe-PI to Fe-graphene with improved tribological properties. Consequently, the friction coefficients of SMLG/PI and SSLG/PI are lower than those of LSLG/PI and LMLG/PI. The interlayer van der Waals forces of SMLG are weaker than those of SSLG with respect to interfacial friction, leading to a lower friction coefficient [26]. This response can be rationalized by the structural discrepancy between SMLG and SSLG. As multi-layer graphene, SMLG exhibits weaker interlayer van der Waals forces and is more susceptible to interlayer sliding under shear stress. In contrast, SSLG, as a single-layer graphene, mainly undergoes direct interfacial shearing at the graphene-Fe interface. The facile interlayer sliding of SMLG effectively lowers the shear resistance, resulting in a smaller friction coefficient. Consequently, the friction coefficient at the interface between Fe and SMLG-modified PI is lower than that between Fe and SSLG-modified PI.

At the same filler content, the large-size morphologies of LMLG and LSLG provide less extensive dispersion in the PI matrix, which lowers the probability of participating in interfacial friction (Figure 11d). As such, the beneficial effect of the multi-layer in reducing friction is not effectively exerted. For graphene with the same flake size, the PI unit cell modified with LSLG has a more compact internal structure (Figure 11e) and a higher binding energy with PI than that modified with LMLG. This effectively constrains the mobility of polymer chains: under shear stress, more PI molecular chains are tightly held on the graphene surface via electrostatic and van der Waals forces, which weakens the interaction between the Fe atoms of the friction pair and PI molecules and results in a lower friction coefficient. Therefore, the friction coefficient of LSLG/PI is lower than that of LMLG/PI.

These results demonstrate that at a given filler loading, the tribological properties of PI composites can be precisely regulated by optimizing the combined morphology of the graphene fillers, including flake size and layer number. The SMLG/PI composite with optimal lubrication performance is suitable for precision bearings, linear guides, and compressor piston rings, where low friction and wear resistance are critical for improving energy efficiency and component longevity.

### 3.5. Atomic Concentration

**Figure 12:**
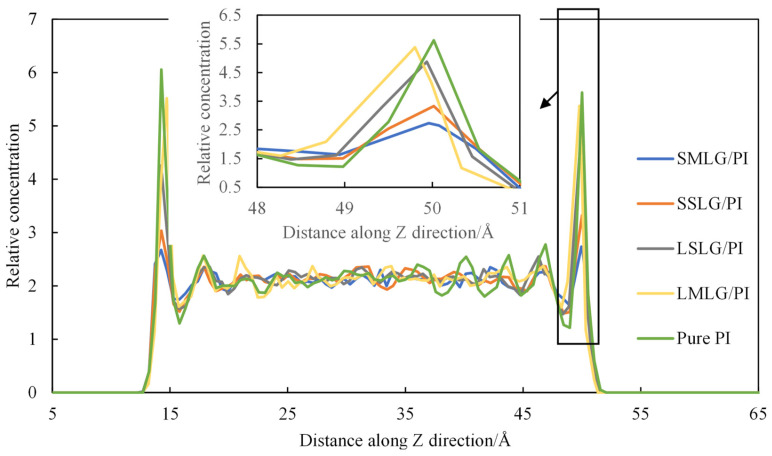
Variation in the atomic concentrations of PI and PI modified with graphene along the Z direction.

An atomic concentration maximum is observed at approximately 1.2 nm and 5.0 nm along the thickness direction, which coincides with the friction interface, as shown in Figure 12. The high relative atomic concentration at this location indicates enhanced molecular mobility, implying that the intermolecular interactions at the friction interface are stronger than those present in the middle region of the matrix. At the friction interface, the atomic concentration is positively correlated with the friction coefficient: a higher atomic concentration results in a larger contact area and, consequently, a higher friction coefficient [19,30].

As illustrated in Figure 12, the atomic concentration of graphene-modified PI is lower than that of pure PI during friction. This phenomenon is attributed to the reinforcing effect of graphene, where PI adsorbs on the graphene surface, forming a denser molecular structure. This compact structure serves to maintain the integrity and stability of the matrix unit cell, improving the tribological performance of the composite. The relative atomic concentration increases in the sequence, SMLG/PI < SSLG/PI < LSLG/PI < LMLG/PI < pure PI at the friction interface. When compared with pure PI, the maximum atomic concentrations of the modified PI composites are reduced by 54.8%, 45.0%, 19.4%, and 8.9%, respectively. This trend is consistent with the variation in tribological performance, suggesting that the simulation results are reliable.

The atomic concentration of PI modified with small-sized graphene (SMLG and SSLG) is lower than the large-sized graphene systems (LSLG and LMLG). At a fixed filler loading, the smaller SMLG and SSLG flake size is beneficial in terms of migration to the friction interface. The smaller flakes can more effectively interact with PI at the friction interface, limiting the extent of molecular motion. Moreover, the small-sized graphene participates in the tribological response and interaction with the friction pair, limiting direct involvement of PI in the friction process. At the fixed filler loading, SMLG exhibits superior tribological performance to SSLG, accompanied by impeded PI molecular motion. Consequently, the atomic concentration of SMLG/PI at the friction interface is lower than that of SSLG/PI.

In contrast, LSLG and LMLG, with larger flake sizes, have a lower probability of participating in interfacial friction and are farther from the friction interface. The PI adsorption capacity at the friction interface is limited, failing to effectively inhibit molecular motion at the interface or sufficiently maintain the stability and integrity of the matrix. At the fixed filler loading, LSLG exhibits more uniform dispersion, a higher binding energy with PI, and closer proximity to the friction surface compared with LMLG. The stronger adsorption of PI molecules at the friction surface better ensures structural integrity and stability. Consequently, the atomic concentration of LSLG/PI at the friction interface is lower than that of LMLG/PI.

### 3.6. Temperature Distribution

**Figure 13:**
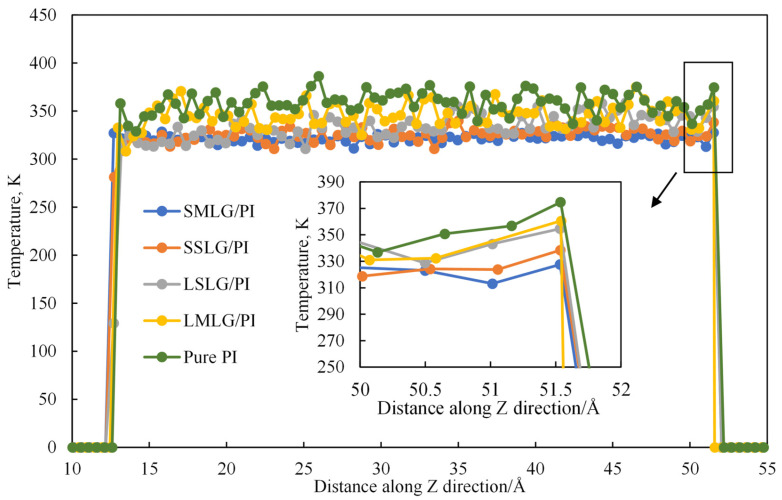
Variations in the temperature of PI and PI modified with graphene along the Z direction.

The temperature distribution of PI modified with graphene of different sizes and number of layers along the Z direction has been examined; the results are shown in Figure 13. The peak temperatures occur at the interface contact surface (at approximately 1.2 nm and 5.0 nm). The peak temperatures at the friction interface increase in the order: SMLG/PI (331 K) < SSLG/PI (340 K) < LSLG/PI (359 K) < LMLG/PI (375 K) < pure PI (386 K). When compared with pure PI, the peak temperatures of the composites are reduced by 14.4%, 12.0%, 7.1%, and 2.9%, respectively, which is consistent with the trend observed in the friction analysis. A lower peak temperature at the friction interface suggests less frictional heat generation, better lubrication and heat dissipation, resulting in superior tribological performance [19,31].

Graphene exhibits good thermal conductivity [32], allowing a rapid transfer of frictional heat. At the same filler content, the small-sized graphene (SMLG and SSLG) acts to more evenly disperse the heat generated relative to large-sized LMLG and LSLG. During friction, heat transfer along the thickness direction is more uniform in the case of SMLG/PI and SSLG/PI, resulting in lower peak temperatures than observed for LSLG/PI and LMLG/PI. The friction coefficients decrease successively in going from pure PI to LMLG/PI, LSLG/PI, SSLG/PI, and SMLG/PI, resulting in lower friction energy and less heat generated. In particular, at the same filler content, SMLG provides more uniform heat transfer; meanwhile, interlayer shear within its multi-layer structure may dissipate part of the frictional energy. As a result, SMLG/PI exhibits the most uniform temperature distribution and the lowest peak temperature. At the same filler content, LMLG/PI shows the poorest friction performance for the composites. The greater associated distance from the friction surface results in less efficient heat conduction, resulting in a higher interface temperature compared with SMLG/PI, SSLG/PI, and LSLG/PI.

## 4. Conclusions

In this study, molecular dynamics simulations were used to qualitatively investigate the combined morphology-dependent effects of graphene flake size and layer number on the mechanical and tribological properties of modified PI at a fixed filler mass fraction. The results indicate that graphene incorporation can reduce the FFV of PI and improve its mechanical and tribological responses.

At an identical filler content, the four combined graphene morphologies exhibit distinct reinforcement effects. Among the morphologies examined, the combination of smaller flakes and fewer layers (SSLG) produces more uniform dispersion, higher interfacial binding energy, and a lower FFV (24.6% lower than that of neat PI) and stronger interfacial interaction, leading to a higher elastic modulus (a 70.4% increase). The FFV values of SMLG/PI, LSLG/PI, and LMLG/PI decrease by 24.1%, 21.1%, and 10.8%, respectively, while their elastic moduli increase by 45.2%, 26.5%, and 14.0%, respectively.

During friction, graphene restricts the movement of PI chains via van der Waals forces and weakens the contact between PI and metallic friction pairs, thereby reducing the friction coefficient. Smaller flakes facilitate graphene migration to the friction interface, while the multi-layer structure enables interlayer sliding; these two morphological factors jointly determine the tribological performance of the composites. SMLG/PI exhibits the best friction-reducing ability, with its friction coefficient, interfacial molecular concentration, and peak temperature decreasing by 48.3%, 54.8%, and 14.4%, respectively, compared with neat PI. For SSLG/PI, LSLG/PI, and LMLG/PI, the friction coefficients decrease by 32.0%, 23.2%, and 10.3%, respectively, while the interfacial molecular concentrations decrease by 45.0%, 19.4%, and 8.9%, respectively, and the peak temperatures decrease by 12.0%, 7.1%, and 2.9%, respectively.

Future work will expand graphene size/layer variables, construct aligned graphene models, and study functional fillers under high-temperature oxidation. Using the atomic interfacial parameters derived herein, we will integrate coarse-grained molecular dynamics and finite element multiscale coupling to build models covering micron-sized fillers for more realistic engineering simulations. This study offers qualitative comparative trends rather than absolute constants, guiding the custom design of graphene/PI composites for optimized mechanical and tribological performance.

## Figures and Tables

**Table 1:** Mechanical properties of PI and PI modified with graphene of different sizes and number of layers (GPa).

System	*E*_1_	*E*_2_	*E*_3_	*E*_aver_	*B*_R_	*B*_V_	*B*_H_	*G*_R_	*G*_V_	*G*_H_
Pure PI	3.13	3.28	3.22	3.21	4.14	4.25	4.20	1.29	1.34	1.32
SSLG/PI	6.00	5.20	5.20	5.47	7.76	8.01	7.88	1.67	2.08	1.88
LSLG/PI	3.86	4.34	3.99	4.06	5.17	5.31	5.24	1.46	1.50	1.48
SMLG/PI	4.90	4.54	4.54	4.66	7.49	7.65	7.57	1.44	1.60	1.52
LMLG/PI	3.75	3.72	3.50	3.66	4.85	5.00	4.93	1.42	1.48	1.45

**Table 2:** Comparison of the elastic modulus in the Y direction obtained from the constant stress and constant strain methods.

System	*E*_2_-Constant Strain (GPa)	*E*_2_-Constant Stress (GPa)	Error
Pure PI	3.28	3.02	7.9%
SSLG/PI	5.20	5.49	5.6%
LSLG/PI	4.34	4.01	7.6%
SMLG/PI	4.54	4.41	2.9%
LMLG/PI	3.72	3.67	1.3%

**Table 3:** Friction coefficients of pure PI and PI modified with graphene.

System	Friction Coefficients
Pure PI	0.203
SSLG/PI	0.138
LSLG/PI	0.156
SMLG/PI	0.105
LMLG/PI	0.182

## Data Availability

The original contributions presented in this study are included in the article. Further inquiries can be directed to the corresponding author.
